# Can polyphenols improve the gut health status in pre-clinical study with diet-induced obesity? A protocol for systematic review and/or meta-analysis

**DOI:** 10.1097/MD.0000000000028162

**Published:** 2021-12-10

**Authors:** Lêda Karla Monteiro Dias, Gidyenne Christine Bandeira Silva de Medeiros, Ana Karolinne Nascimento Silva, Ana Heloneida de Araujo Morais, Juliana Kelly da Silva-Maia

**Affiliations:** a Nutrition Postgraduate Program, Health Sciences Center, Federal University of Rio Grande do Norte, Natal, Brazil; b Department of Nutrition, Federal University of Rio Grande do Norte, Natal, Rio Grande do Norte, Brazil; c Biochemistry and Molecular Biology Postgraduate Program, Center for Biosciences, Federal University of Rio Grande do Norte, Natal, Brazil.

**Keywords:** dysbiosis, gut health, microbiota, obesity, polyphenols

## Abstract

**Introduction:**

Obesity is characterized as a low-grade inflammation that impairs physiological functions, including intestinal functioning and gut microbiota balance. Dietary polyphenols can be a strategy for obesity management, collaborating to preserve or recover gut health through antioxidant and anti-inflammatory actions, as well as modulators of the microbiota. This study describes a systematic review protocol to elucidate effects of polyphenols on intestinal health of pre-clinical models with diet-induced obesity. AIM: Our aim is to evaluate evidence about polyphenols’ effects in the gut microbiota composition and diversity, parameters of the physical and molecular status of the gut barrier in obese models, additionally, understand the possible involved mechanisms.

**Methodology:**

A protocol was developed and published on PROSPERO (Registration No: CRD42021262445). Preferred Reporting Items for Systematic Review and Meta-Analysis Protocols is used to outline the protocol. The articles will be selected according to the PICOS strategy (population, interventions, control, outcome, and study design) in the following databases: PubMed, Science Direct, Scopus, Web of Science, and EMBASE. Experimental studies performed on rats and mice with a control group that describes treatment with polyphenols (from food matrix or crude extracts or isolated compounds) at any frequency, time, and dose will be included. Two reviewers will, independently, select the papers, extract data, and evaluate the data quality. The Systematic Review Center for Laboratory Animal Experimentation (SYRCLE) tool will be used to assess the risk of bias.

**Expected results:**

Results will be showed through of native synthesis and, if possible, a metanalysis will be conducted. The review produced with this protocol can show the scientific evidence level about polyphenols’ effects in intestinal health in obesity status.

## 1 Introduction

Bioactive compounds, such as polyphenols, are found in different food sources and can modulate inflammation and oxidative stress by acting in metabolic vias from obesity and other noncommunicable diseases.^[1]^

Polyphenols are secondary metabolites from vegetables with key roles as a defense against microorganisms, insects, and environmental stress.^[2]^ Furthermore, these compounds have therapeutic actions such as antimicrobial, antiproliferative, antioxidant, and anti-inflammatory, which can act in local and systemic sites.^[3,4]^ From those functional properties, polyphenols have been suggested as adjuvants in obesity treatment.^[5–7]^

Nowadays, obesity is a major and growing public health problem worldwide. According to the World Health Organization, 39% of adults were overweight and 13% were obese.^[8]^ This is a multifactorial and complex disease with an excessive accumulation of adipose tissue. There is evidence of correlation among gut microbiota disbalance, physiopathology process, and metabolic disturbers present on obesity.^[9,10]^

Intestinal dysbiosis can be associated with low-grade inflammation from obesity that induces significant modifications to the gut microbiome. Eutrophic individuals show more diverse gut microbiota than those with fat tissue accumulation, which can contribute to metabolic disturbs through an axis of communication with adipose tissue.^[11,12]^ The dominative gut microbial phyla are Firmicutes, Bacteroidetes, Actinobacteria, Proteobacteria, and changes in the diversity and abundance of microorganisms impact health. Changes in the gut microbiota composition in obese individuals have been found, with a higher ratio of *Firmicutes* to *Bacteroidetes* as well as a reduced diversity of microbiota in the gut compared with eutrophic individuals.^[13]^ The imbalance between pathogenic and nonpathogenic microorganisms in the gut impairs the epithelial barrier and intestinal functions, that is, cause lower nutrient absorption, higher permeability to bacteria cytokines, and induce inflammatory response.^[14]^

Sequentially, gut microbiota unbalance impairs the production of short-chain fatty acids (SCFA), which are yielded by metabolization of fermentable and non-digested compounds as saccharides, including glycosylated phenolic compounds. Examples of bacteria species with this role are *Lactobacillus, Lachnospiraceae,* and *Ruminococcacea*e.^[15]^ SCFA are key to maintaining intestinal health because are a fuel source for intestine epithelial cells, preserving the gut barrier integrity, besides reducing the intraluminal pH that avoids the pathogenic bacteria growing and stimulating the mucus production by caliciform cells. Moreover, impaired intestinal barrier increases the risk of bacteria translocation and other harmful substances leading to the production of inflammatory cytokines and contributing to keeping chronic inflammations as in obesity.^[16]^

Unbalanced diet composition can contribute to obesity perpetuation and intestinal health disturbs. On the other hand, a healthier diet can be a strategy to provide bioactive compounds, like polyphenols, protecting against diseases. Indeed, diet interventions have been broadly studied as determinant factors to intestinal health and microbiota modulation. Highlighting that intestinal health is referred, besides to microbiota balance, to mucosal barrier integrity and function, that is, preserved processes as digestion, absorption, and host defense.^[4]^ In this sense, polyphenols are possible therapeutic approaches to prevent and treat obesity and the associated dysbiosis, due to their capacity to modulate the gut microbiota and intestinal permeability, as well as control inflammatory and oxidative processing.^[17]^ Additionally, the microbiota composition can influence polyphenols’ effects because the produced metabolites through these compounds depend, in part, on gut microbiota metabolism.^[13]^

Preclinical experiments with animal models are important to elucidate the effects and involved mechanisms in different situations, which can be tricky in clinical experiments once required more invasive procedures as biopsies. Data from experimental studies bring much information that are essentials to clinical research advances.^[18]^ In this regard, a systematic review gathering data from preclinical studies that assessed the bioactive compounds’ effects and mechanisms in obesity-associated dysbiosis and other parameters about health intestinal is necessary to point the scientific evidence level and direct the future clinical experiments and applications.^[16,17,19]^

Therefore, the review that will be produced with this protocol aims to show the scientific evidence about polyphenols’ effects in intestinal health in obesity status, and possible action mechanisms as agents with antioxidant, anti-inflammatory, and of balance microbiota properties.

## 2 Methods

### 2.1 Protocol and registration

The protocol was prepared according to the Preferred Reporting Items for Systematic Reviews and Meta-Analyses Protocols (PRISMA-P).^[20]^ This protocol was registered in the International Prospective Register of Systematic Reviews (PROSPERO) on August 27, 2021 with registration number CRD42021262445, and available at https://www.crd.york.ac.uk/prospero/display_record.php?RecordID=262445. Ethical approval is not necessary for this research, because it is a review study with the use of secondary data.

**Figure 1:**
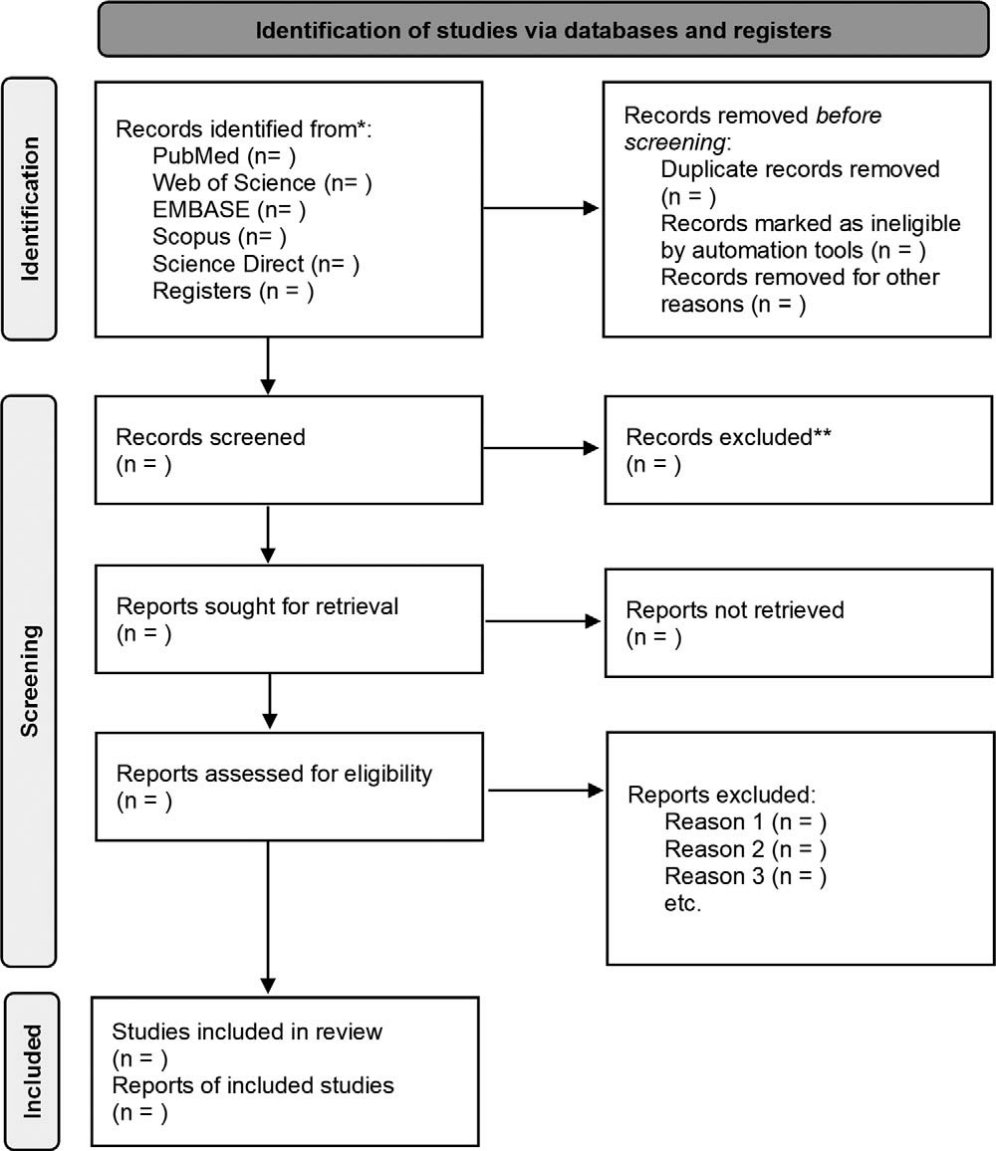
Article selection flowchart. Adapted from Preferred Reporting Itens for Systematic Reviews.

The systematic review will follow the Preferred Reporting Items for Systematic Reviews and MetaAnalyses (PRISMA).^[21]^ The flowchart was created to demonstrate the selection of study articles PRISMA (Fig. 1). The Preferred Reporting Items for Systematic Review and Meta-Analysis Protocols (PRISMA-P) checklist^[22]^ was applied to improve the quality of the systematic review data.

### 2.2 Review question

Can polyphenols improve the gut health status in pre-clinical studies with diet-induced obesity?

### 2.3 Eligibility criteria

Original articles must attend the eligibility criteria according to the PICOS (population, interventions, control, outcomes, and study design) (Table 1).

**Table 1:** Elements of the research question according to the PICOS strategy.

Description	Abbreviation	Elements of the question
Population	P	Rats (Rattus norvegicus) and mice (Mus musculus) of both sexes and ages with diet-induced obesity.
Interventions	I	Treatment with isolated polyphenols or extracts with identified polyphenols.
Control/comparison	C	Rats (Rattus norvegicus) and mice (Mus musculus) with diet-induced obesity without treatment.
Outcomes	O	Markers associated with gut health status and microbiota intestinal.
Study design	S	In vivo experimental studies

### 2.4 Inclusion criteria

This review will include experimental studies with rats or mice of both sexes and ages, evaluating the effect of polyphenols in the treatment of diet-induced obesity, regarding the action in the intestinal health. The models must be treated with isolated polyphenols or crude extracts with identified polyphenols or food matrix with identified polyphenols. All types of routes and doses of administration will be included.

### 2.5 Exclusion criteria

Documents that are not scientific articles, case reports, reviews, studies with other animal models. Studies without polyphenols identification or studies that combine treatment with polyphenols and antibiotics, probiotics, or prebiotics. Studies that do not time of the experiment, frequency, and doses administered, and studies without a control group. Studies that do not investigated effects in intestinal health will be excluded.

### 2.6 Search strategy

Searches will be conducted in the following databases: PubMed; Science Direct; Scopus; Web of Science; BVS and EMBASE by 2 independent researchers (L.K.M.D and A.K.N.S.). The full search strategy will be based on the search components: polyphenols OR flavonoids OR phenols OR anthocyanins; obesity OR adiposity; rats OR mice; microbiota OR microbiome OR disbiosis. The final search strategy will be tailored using Medical Subject Headings (MeSH) terms, entry terms, and synonyms linked with Boolean operators.

### 2.7 Selection process

The list of references of all studies included in the systematic review will be screened. Before final analysis, the searches will be updated. No restrictions will be applied to language and year of publication, or geographic limits.

All articles by the search strategy must be exported to the Rayyan QCRI application (The Systematic Reviews Web).^[23]^ Duplicated studies will be removed, following, then, the initial selection by considering their titles, abstract, and keywords. Discrepancies between the reviewers will be identified and resolved through consensus, and a third reviewer (J.K.SM) will be involved when necessary to decide whether to include the study. Studies excluded will be recorded, and their reason for exclusion will be reported in the review. All researchers will then review the full text of all studies considered eligible for inclusion. The management of references will be applied to the software Mendeley.^[24]^ In the first step of data synthesis, we will present the results of the study selection process using the PRISMA statement flowchart.^[25]^

### 2.8 Types of results

The primary outcomes include:

1. Effect of the polyphenol(s) in the gut microbiota modulation: Effect in the markers of the intestinal microbiota diversity (colony-forming unit—CFU or Operational taxonomic units—OTUs; continuous);
2. Effect of the polyphenol(s) in the gut health status: Short-chain fat acids—butyrate, acetate, and propionate (mmol/g; continuous); Tight junction expression—occludin and claudin (multiple units; continuous). Morphological analysis from intestinal barrier (dichotomous). Gene expression and dosage of inflammatory cytokines in gut level such as IL-1, IL-6, and TNF-α (pg/mL; continuous).

### 2.9 Data extraction

The extraction of data will be standardized and performed by 2 independent authors (L.K.M.D and A.K.N.S.), creating a database in a pre-designed and previously tested spreadsheet in the Excel program. The third author (J.K.SM) will verify the discrepancies and organize the information to construct the original text. The following information will be presented in this database: article identification, experimental design, polyphenol(s) administrated, number of experimental groups and duration of follow up, method of group allocation, animal model data (species, sex, weight, age, and oral diet information), type of route of administration, dosage, time of exposure, and outcomes, mainly, around possible action of polyphenols in the gut health.

### 2.10 Risk of bias and quality assessment

The Systematic Review Center for Laboratory Animal Experimentation (SYRCLE) tool will be used to assess the risk of bias. Two evaluators will perform the independent readings. Discrepancies will be remedied with a third appraiser.

### 2.11 Data synthesis and analysis

At the end of the analysis of the data extracted from the included studies (at least two), if clinical and/or methodological and/or statistical homogeneity are found, a meta-analysis will be performed. This will be carried out using the Rev Man Analyses statistical package in Review Manager v.5.3. If a high level of heterogeneity (>50%) will found in the studies, we will explore the heterogeneity by subgroup analysis or meta-regression.

Because of the exploratory nature of animal studies, the random effects model will be used to account for anticipated heterogeneity. For dichotomous outcomes, we will derive the OR and 95% CI for each study. The heterogeneity between the trial results will be evaluated using a standard *I*^2^ test with a significance level of *P* .1, and the *I*^2^ statistic, which is a quantitative measure of inconsistency across studies, with a value of 0% indicating no observed heterogeneity, and values of 50% indicating substantial levels are present. If there is heterogeneity (*I*^2^ 75%), a random-effects model will be used to combine the trials to calculate the relative risk (RR) and 95% CI, using the DerSimonian-Laird algorithm in meta for package, a meta-analysis package for R. Other study characteristics and results will be summarized narratively if a meta-analysis cannot be performed for all or some of the included studies. If possible, funnel plots will also be used to assess the presence of potential reporting biases, and a linear regression approach will be used to evaluate funnel plot asymmetry. A linear regression approach will be used to evaluate funnel plot asymmetry. Adjustments will be performed, if needed in the studies.

### 2.12 Subgroup analysis

Subgroup analyses will be performed if relevant data are available. If possible, the following will be undertaken: species (stratified per species); sex (stratified per sex); duration of treatment; dose; type of polyphenol used.

## 3 Ethics and dissemination

Ethical approval and informed consent are not necessary for this research because it is a systematic review (use of secondary data).

## 4 Discussion

This protocol article describes the development of a systemic review based on the hypothesis that bioactive compounds, specifically polyphenols, can act in obesity management. Contributing positively to gastrointestinal function, gut microbiota balance, and mitigating inflammation. Although there are published systematic reviews regarding polyphenols impact on intestinal health, they do not approach the obesity influence.^[16,26,27]^ On the other hand, reviews about polyphenols action on obesity treatment do not elucidate the effects on gut health.^[28,29,30]^ In this sense, our proposed review is innovative and will fill the lacuna of scientific evidence about polyphenols’ properties to improve the intestinal health impaired by obesity. These data could address future investigations and the application of polyphenols in clinical practice.

The first findings of the relationship between obesity and intestinal functions were shown by preclinical studies with animal models. Hence, scientific investigations with experimental models are important to better understand this subject and providing data to directing future clinical studies, and, therefore, contributing to new treatments developing to several diseases.^[18,31]^ Li et al showed that gastrointestinal inflammation and alterations in inflammatory markers in mice with diet-induced obesity. This studied found increased expression of TNF-α in adipose tissue and liver, IL-1β in the proximal colon, and higher permeability of intestinal barrier to proinflammatory cytokines.^[32]^

Although it is not totally understood the mechanisms, gut microbiota imbalance alters the organism's homeostasis and contributes to metabolic disturbances associate with obesity.^[11]^ Current studies have shown that obese organisms have a higher prevalence of Firmicutes than Bacteroidetes, the opposite is observed in eutrophic individuals. These changes in microbiota composition result in high absorption, fat deposition on the liver, decreasing of anorexigenic hormones, increasing of proinflammatory cytokines, and gut barrier damage.^[33–36]^ Corroborating, dietetic interventions increased Bifidobacteria and reduced weight gain and inflammation, improving the gut barrier function.^[37]^ Accordingly, the intestinal microbiota is an important biomarker, mediator, and therapeutic target in metabolic diseases, including obesity. In this sense, it is relevant to investigate the bioactive compounds as emergent strategies to microbiota modulation and maintenance of intestinal health, aiming prevention and treatment of obesity.

Antioxidant and anti-inflammatory properties, as well as microbiota modulators, are properties from polyphenols, which increases the interest in these compounds as a strategy in obesity treatment.^[38–40]^ Moreover, the use of the conventional drugs to treat obesity is limited by no specificity and side effects of nowadays available medicines.^[41]^

Polyphenols can be metabolized by gut bacteria stimulating the growth of beneficial species as Lactobacillus and Bifidobacteria, and reducing the pathogenic ones as coliforms.^[42]^ These compounds act also in control of the inflammatory pathways, reducing the proinflammatory cytokines as LPS, TLR, TNF, IL-1, and IL-1R.^[43]^ A recent study showed that Wistar rats fed with a high-fat diet and treated with grape marc had significant improvements since weight loss, control of glycemia and lipid, until better microbiota profile, with high ratio of *Lactobacillus* spp. and *Bacteroidetes* spp.^[44]^ Other work with blueberry has found similar results, showing gut microbiota modulation, improvement of barrier integrity (increased villosities and mucin and β-defensin-2 expression), attenuating obesity-associated inflammation (lower proinflammatory cytokines expression), and better response to insulin in rats with diet-induced obesity.^[45]^

Therefore, it is suggested that polyphenols can be applied to the treatment of obesity-associated inflammatory processes and maintenance of intestinal health. However, literature data about polyphenols’ effects on obesity and intestinal health are fragmented challenging to affirm their efficacy and action mechanisms. This way, it is necessary to realize a systematic review focusing on the polyphenols action on intestinal health concomitantly with obesity treatment. The present protocol will address a systematic review that can cover the aforementioned gaps, consequently, direct future works and application of polyphenols in obesity treatment.

## Acknowledgments

This work was supported by the Coordenação de Aperfeiçoamento de Pessoal de Nível Superior (Finance Code 001 – CAPES),

## Author contributions

**Conceptualization:** Lêda Karla Monteiro Dias, Ana Heloneida de Araujo Morais, Juliana Kelly Silva-Maia.

**Data curation:** Lêda Karla Monteiro Dias, Gidyenne Christine Bandeira Silva de Medeiros, Ana Karolinne Nascimento Silva, Ana Heloneida de Araujo Morais, Juliana Kelly Silva-Maia.

**Formal analysis:** Lêda Karla Monteiro Dias, Gidyenne Christine Bandeira Silva de Medeiros, Ana Karolinne Nascimento Silva, Ana Heloneida de Araujo Morais, Juliana Kelly Silva-Maia.

**Investigation:** Lêda Karla Monteiro Dias, Gidyenne Christine Bandeira Silva de Medeiros, Ana Karolinne Nascimento Silva, Ana Heloneida de Araujo Morais, Juliana Kelly Silva-Maia.

**Methodology:** Lêda Karla Monteiro Dias, Gidyenne Christine Bandeira Silva de Medeiros, Ana Karolinne Nascimento Silva, Ana Heloneida de Araujo Morais, Juliana Kelly Silva-Maia.

**Project administration:** Ana Heloneida de Araujo Morais, Juliana Kelly Silva-Maia.

**Resources:** Juliana Kelly Silva-Maia.

**Supervision:** Gidyenne Christine Bandeira Silva de Medeiros, Ana Heloneida de Araujo Morais, Juliana Kelly Silva-Maia.

**Validation:** Lêda Karla Monteiro Dias, Gidyenne Christine Bandeira Silva de Medeiros, Ana Heloneida de Araujo Morais, Juliana Kelly Silva-Maia.

**Writing – original draft:** Lêda Karla Monteiro Dias, Gidyenne Christine Bandeira Silva de Medeiros, Ana Karolinne Nascimento Silva, Ana Heloneida de Araujo Morais, Juliana Kelly Silva-Maia.

**Writing – review & editing:** Lêda Karla Monteiro Dias, Gidyenne Christine Bandeira Silva de Medeiros, Ana Karolinne Nascimento Silva, Ana Heloneida de Araujo Morais, Juliana Kelly Silva-Maia.
